# *Operando* X-ray scattering study of thermoelectric β-Zn_4_Sb_3_

**DOI:** 10.1107/S205225251901580X

**Published:** 2020-01-01

**Authors:** Lasse Rabøl Jørgensen, Christian Moeslund Zeuthen, Kasper Andersen Borup, Martin Roelsgaard, Nils Lau Nyborg Broge, Jonas Beyer, Bo Brummerstedt Iversen

**Affiliations:** a Department of Chemistry, University of Aarhus, Langelandsgade 140, Aarhus C 8000, Denmark

**Keywords:** thermoelectric materials, zinc antimonides, pair distribution function, *operando* study, powder X-ray diffraction, electrical resistance

## Abstract

The application of thermoelectrics for energy harvesting depends strongly on operational reliability and it is therefore desirable to investigate the structural integrity of materials under operating conditions. We have developed an *operando* setup capable of simultaneously measuring X-ray scattering data and electrical resistance on pellets subjected to electrical current. Here, *operando* investigations of β-Zn_4_Sb_3_ are reported at current densities of 0.5, 1.14 and 2.3 A mm^−2^. At 0.5 A mm^−2^ no sample decomposition is observed, but Rietveld refinements reveal increased zinc occupancy from the anode to the cathode demonstrating zinc migration under applied current. At 1.14 A mm^−2^ β-Zn_4_Sb_3_ decomposes into ZnSb, but pair distribution function analysis shows that Zn_2_Sb_2_ units are preserved during the decomposition. This identifies the mobile zinc in β-Zn_4_Sb_3_ as the linkers between the Zn_2_Sb_2_ units. At 2.3 A mm^−2^ severe Joule heating triggers transition into the γ-Zn_4_Sb_3_ phase, which eventually decomposes into ZnSb, demonstrating Zn ion mobility also in γ-Zn_4_Sb_3_ under electrical current.

## Abstract 2

A thermoelectric *operando* setup for synchrotron X-ray scattering has been developed in order to study the operational stability of thermoelectric materials in real-world conditions.

## 1. Introduction   

Thermoelectric (TE) materials are capable of interconverting thermal and electrical energy and they provide exciting opportunities for green harvesting of the vast amounts of waste heat released *e.g.* in engines or industrial processes. Many TE materials are explored for power-generation applications, such as silicides (Sadia *et al.*, 2016 ▸), PbTe (Pei *et al.*, 2011 ▸), clathrates (Johnsen *et al.*, 2006 ▸), antimonides (Pedersen *et al.*, 2007 ▸) and half-Heuslers (Appel & Gelbstein, 2014 ▸). Apart from the obvious need for high performance, low cost and environmentally friendly materials, one of the main obstacles of TE technology is the lack of material stability during operation at high temperature with the material subjected to large temperature gradients and electrical current. Material degradation has been encountered not only in recent high-performance materials such as SnSe, Cu_2_Se and Zn_4_Sb_3_ (Li *et al.*, 2016 ▸; Brown *et al.*, 2014 ▸; Hung *et al.*, 2017 ▸; Yin *et al.*, 2010 ▸), but even in inorganic clathrates usually considered as extremely stable compounds (Reardon *et al.*, 2017 ▸). TE energy harvesting relies strongly on optimizing operational reliability without compromising energy-conversion efficiency.

Studies of the stability of TE materials typically use experimental conditions that do not resemble an operating module. This primarily involves powder X-ray diffraction (PXRD) studies at elevated temperatures, or thermal cycling, using various atmospheres (Jørgensen *et al.*, 2018*a* ▸, 2018*b* ▸; Pedersen & Iversen, 2008 ▸; Pedersen *et al.*, 2006 ▸, 2010 ▸; Fischer *et al.*, 2018 ▸; Reardon *et al.*, 2017 ▸; Yin *et al.*, 2010 ▸). Some studies have exposed TE materials to (semi)realistic conditions but they have not simultaneously characterized the structural or TE properties (*operando*) (Reardon *et al.*, 2017 ▸; Hung *et al.*, 2017 ▸; Yin *et al.*, 2014 ▸). *Operando* studies are well established *e.g.* for battery materials (Senyshyn *et al.*, 2012 ▸; Ulvestad *et al.*, 2015 ▸; Taminato *et al.*, 2016 ▸) or catalysts (Topsøe, 2003 ▸). Indeed, *operando* characterization is invaluable to battery research, where atomistic understanding of ion diffusion and structural stability during (dis)charging cycles is essential for improving battery technology (Nelson *et al.*, 2012 ▸; Cuisinier *et al.*, 2013 ▸). Here we introduce an experimental setup capable of mimicking real-life TE conditions. By using high-energy synchrotron radiation (60 keV) to penetrate samples it allows characterization of both atomic structure and electrical resistance of densified bulk samples exposed to direct current.

The effect of electrical current on TE materials is rarely investigated in stability studies but it is particularly important for mixed ionic electronic conductors such as Cu_2_Se and β-Zn_4_Sb_3_. In these materials, the coexistence of a rigid anion crystal structure and a liquid-like cation sublattice causes the materials to possess a low thermal conductivity and a relatively high electrical conductivity making them highly suitable as TE materials (Caillat *et al.*, 1997 ▸). Conversely, having highly mobile ions in the structure may not be ideal for reliable long-term operation in electric current. This has been the essential challenge for β-Zn_4_Sb_3_, which has Zn migrating towards the cathode end of the material, that is, in the direction of the current during spark plasma sintering (SPS) (Yin *et al.*, 2014 ▸, 2012 ▸). Since only *ex situ* characterization methods have been employed in studying the Zn migration, the atomistic insight into the process is limited. Here we report an *operando* study of densified rod-shaped samples of β-Zn_4_Sb_3_ subjected to electrical current, and three successful high-energy synchrotron X-ray data collections were achieved. For two current settings the detector was placed to provide PXRD data, while for the third current setting, total scattering data were obtained at short detector distances allowing for subsequent pair distribution function (PDF) analysis.

**Figure 1:**
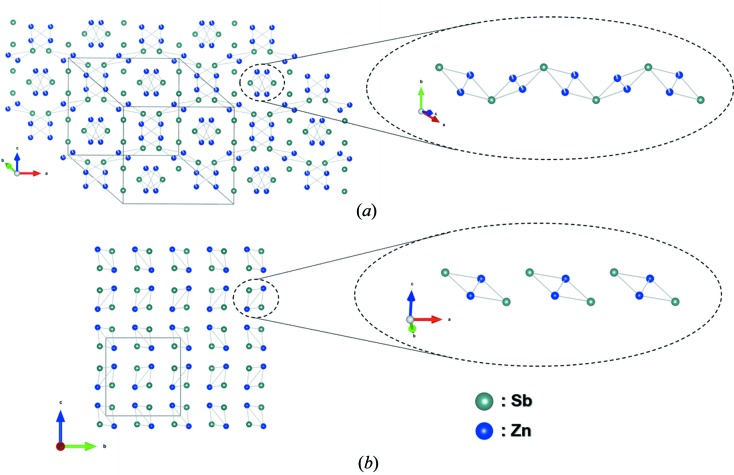
The atomic structure of (*a*) β-Zn_4_Sb_3_ and (*b*) ZnSb. The interstitial zinc sites in β-Zn_4_Sb_3_ are not shown. The right side of the figures shows the rhomboid units present in both structures. The figures were generated using the *VESTA* software (Momma & Izumi, 2011 ▸).

β-Zn_4_Sb_3_ crystallizes in space group 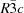 with Zn at the 36*f* site (denoted Zn1), and Sb at the 18*e* and 12*c* sites (denoted Sb1 and Sb2, respectively), Fig. 1 ▸(*a*). The space- and time-averaged crystal structure has three interstitial Zn sites in close proximity to the main Zn1 site [not included in Fig. 1 ▸(*a*)] (Snyder *et al.*, 2004 ▸). The refined stoichiometry of Zn_3.83(4)_Sb_3_ is consistent with the observed mass density, which was not the case for earlier models (Mayer *et al.*, 1978 ▸). However, later studies have revealed a certain compositional freedom in the Zn content (Toberer *et al.*, 2010 ▸). Here we refer to the compound as β-Zn_4_Sb_3_ noting that this does not reflect the true composition.

## 2. Experimental   

**Figure 2:**
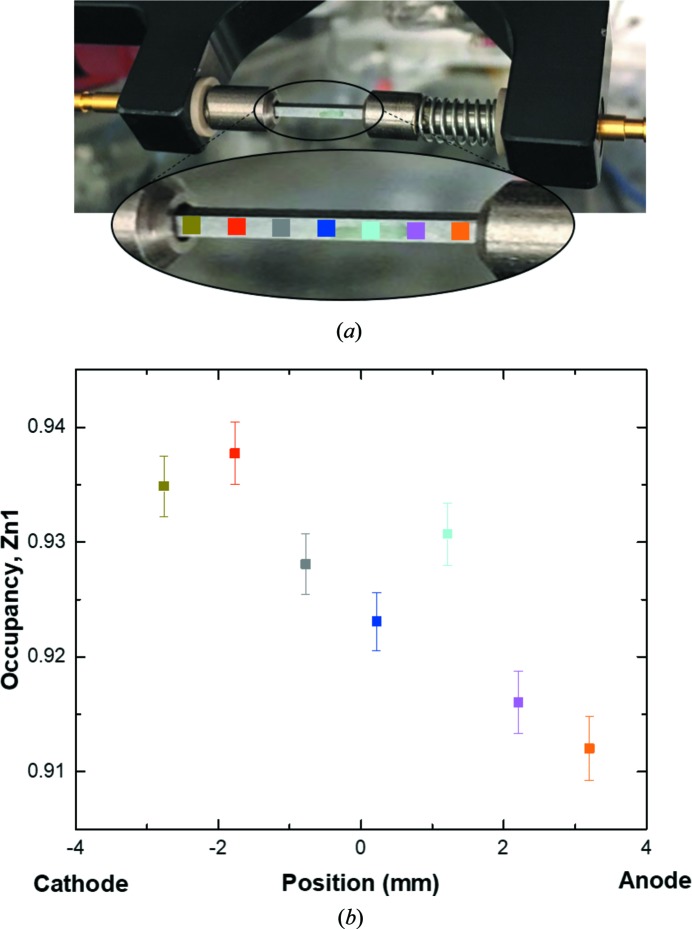
(*a*) The experimental setup installed at beamline P02.1, where the sample is spring loaded between two electrodes and placed in front of the X-ray beam. (*b*) Refined occupancy parameters for the Zn1 site at different positions indicated by the colored squares in (*a*).

The experiments were carried out at beamline P02.1 at PETRA III, DESY, Germany. Because of the high photon energy (60 keV), it is possible to penetrate a densified inorganic sample of 1 mm thickness without extensive absorption (μ*R* < 1.4 for β-Zn_4_Sb_3_, assuming crystallographic density, with μ being the linear absorption coefficient and *R* half the sample thickness). The samples were subjected to direct current by each sample being spring loaded between two electrodes, Fig. 2 ▸(*a*). The current was kept constant during the X-ray experiment. Two molybdenum wires were placed in contact with the sample surface to measure the four point resistance between the two points of contact during the experiment. When measuring total scattering data, a sample-to-detector distance of ∼200 mm was used with the detector placed such that the beam center was at the edge of the detector. This resulted in a maximum *Q* value of ∼33 Å^−1^. For PXRD, the sample-to-detector distance was ∼650 mm to improve angular resolution and the beam center was moved to the center of the detector to increase the detected intensity. The sample-to-detector distances and the instrumental resolution were calibrated using a LaB_6_ NIST sample. The resulting scattering patterns were azimuthally integrated using the *Dioptas* software (Prescher & Prakapenka, 2015 ▸) and the TS data was Fourier transformed in *PDFGetX3* to obtain the PDF (Juhás *et al.*, 2013 ▸). Refinement of PXRD data was carried out using the *FullProf* (Rodríguez-Carvajal, 1993 ▸) and *JANA*2006 (Petrícek *et al.*, 2014 ▸) softwares, and the PDF data was refined using *PDFgui* (Farrow *et al.*, 2007 ▸). A detailed description of the refinement models is given in the Supporting information.

The synthesis of β-Zn_4_Sb_3_ was carried out using the method of Yin *et al.* (2014 ▸). Zinc powder (99.99%, Alfa Aesar) and antimony powder (99.5%, ChemPur) were weighed in a stoichiometric ratio (Zn_4_Sb_3_) and mixed for 15 mins in a ball mill (SpectroMill, Chemplex Industries, Inc.). The powder was then transferred to a graphite die with a 1 inch diameter and compacted using SPS. To compensate for zinc migration caused by the current in the SPS press, a zinc foil (99.95%, Sterilin) of 0.15 mm thickness was placed at the anode of the pellet, resulting in a compositionally homogenous sample as verified by PXRD.

## 3. Results   

The present experiments used 60 keV synchrotron radiation and current densities of 0.5, 1.14 and 2.3 A mm^−2^. These current densities are considerably higher than for operating TE modules but this was necessary because of the limited beam time, and it provides an accelerated stability test. The current densities are similar in magnitude to typical SPS densification processes. The first experiment used 0.5 A mm^−2^ and PXRD data were recorded, see Fig. S1 in the Supporting information. During ∼270 mins no structural transitions were observed. Time-resolved sequential Rietveld refinements were carried out to track changes in unit-cell parameters and atomic displacement parameters (ADPs), and the refined parameters are shown in Fig. S2. Joule heating of the sample causes a rapid increase in both unit-cell parameters and ADPs corresponding to a temperature of ∼65°C immediately after initiating the current exposure. This sample temperature was estimated using the lattice expansion coefficient obtained from a separate variable-temperature PXRD experiment conducted at beamline I15-1 at the Diamond Light Source (Fig. S3). The expansion was found to be 2.15 × 10^−4^ Å K^−1^ and 2.37 × 10^−4^ Å K^−1^ for the *a* axis and *c* axis, respectively. Since the measured sample electrical resistance does not at any time exceed the value measured right after initiating the current (Fig. S1), the sample temperature must reach steady state, and the subsequent relative development of the unit-cell parameters and ADPs is primarily because of structural changes (Zn migration). Similar behaviors are observed for the ADPs of all three atomic sites (Fig. S2), with a linear increase during the entire experiment. The absolute changes in ADPs are largest for the Zn sites. Decreasing the Zn content on the Zn sites, because of migration, will lead to lower average electron density on those sites. Since the present Rietveld model does not vary the site occupancy factor, the effect will be reflected in an increased ADP.

The sample was expected to form a Zn concentration gradient and this was confirmed from refinement of PXRD data collected along the sample with a spatial resolution of 1 mm and sufficient data quality to extract the site occupancy factor for the Zn sites. The obtained occupancy for the Zn1 site is shown in Fig. 2 ▸(*b*), whereas the occupancies of the interstitial Zn sites (Zn2, Zn3, Zn4) are shown in Fig. S4(*b*). The overall composition of the structure is plotted in Fig. S4(*c*). Indeed, the occupancy of the Zn1 site increases when moving from the anode towards the cathode, but this behavior is not seen for the interstitial Zn sites, where the occupancy is approximately constant throughout the sample. The importance of the interstitial sites in the Zn migration process has been suggested but never quantified (Dasgupta *et al.*, 2013 ▸). At first sight, the disordered low-occupancy Zn interstitial sites may be expected to behave somewhat like a liquid, but it appears that it is the main Zn1 that provides ion migration during current exposure. The total Zn content increases from the anode to the cathode, although the absolute values may be inaccurate since the ADPs are fixed to literature values (ICSD No. 159090; Pedersen *et al.*, 2007 ▸). Even so, the relative values are expected to be reliable.

**Figure 3:**
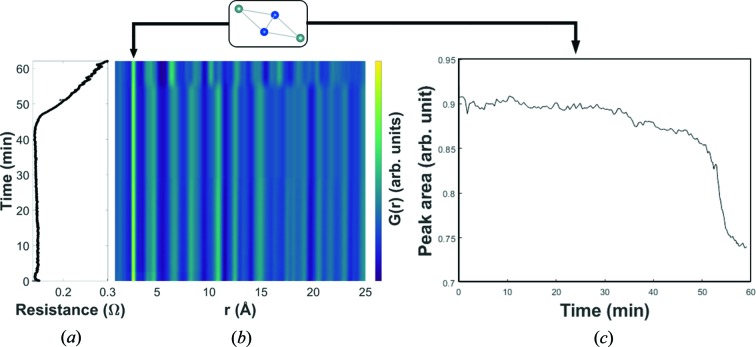
(*a*) Electrical resistivity and (*b*) surface plot of the raw PDF data. (*c*) A single-peak integration of the peak at *r* ≃ 2.7 Å seen in (*b*).

Next, we analyze the PDF data measured with a current density of 1.14 A mm^−2^, Fig. 3 ▸(*b*). After ∼55 mins, an abrupt decomposition of β-Zn_4_Sb_3_ into ZnSb is observed. Even though many of the characteristic distances within the structure change during the decomposition, the shortest distances at *r* ≃ 2.7–2.9 Å are maintained throughout the experiment, indicating that a substructure of β-Zn_4_Sb_3_ is preserved in ZnSb. The *r* ≃ 2.7–2.9 Å correlations originate from rhomboid Zn_2_Sb_2_ units (Fig. 1 ▸) and this motif is present in both β-Zn_4_Sb_3_ and ZnSb with the only difference being the linking of different units by additional Zn in Zn_4_Sb_3_ (absent in ZnSb). The rhomboid unit has been shown to be preserved through the phase transition from the low-temperature α-Zn_4_Sb_3_ structure to the β-Zn_4_Sb_3_ structure (Nylén *et al.*, 2007 ▸). Observing the same unit in ZnSb indicates that the rhomboid Zn_2_Sb_2_ substructure could be a fundamental feature of zinc antimonide structures. To confirm that the correlations at *r* ≃ 2.7–2.9 Å originate from the rhomboid unit, PDFs have been simulated for the Zn_2_Sb_2_ units present in β-Zn_4_Sb_3_ and ZnSb, and compared with the full PDF of β-Zn_4_Sb_3_ and ZnSb, Fig. S7. The rhomboid unit is clearly the main contributor to this correlation.

The integrated intensity of the rhomboid PDF peak as a function of time reveals three distinct regions, Fig. 3 ▸(*c*). Up to ∼30 mins, the intensity decreases only slightly, but in the following 20 mins there is a pronounced decrease. After 50 mins the intensity drops drastically, immediately before the decomposition of β-Zn_4_Sb_3_ at ∼55 mins. A decrease in PDF intensity indicates less correlations at this distance, as expected because of the linking of the rhomboids in β-Zn_4_Sb_3_, which is not present in ZnSb (see Fig. 1 ▸). This is consistent with the refined occupancies in Fig. 2 ▸(*b*), where the Zn1 site becomes depleted because of migration. The present *operando* data suggest that it is the linking Zn1 that leave the β-Zn_4_Sb_3_ structure during decomposition to ZnSb. It is presumably these Zn atoms that migrate during exposure to electrical current leading to a change in the Zn1 occupancy. The difference in slope in the three regions of Fig. 3 ▸(*c*) can be explained by the continuously changing composition, which is known to significantly change the diffusion constant of Zn in β-Zn_4_Sb_3_ (Løvvik *et al.*, 2011 ▸). The electrical resistance is relatively constant during the first 45 mins after which it increases drastically. This increase indicates that the decomposition has occurred at the position of the first resistance probe, which is mounted ∼1 mm from the X-ray beam position. Thus, the decomposition commences in the end of the sample where the current enters, and progresses along the current direction. The ZnSb phase has a higher electrical resistivity compared with that of β-Zn_4_Sb_3_ and will therefore be heated more by the current (Shaver & Blair, 1966 ▸; Caillat *et al.*, 1997 ▸). The continuously increasing resistance reflects that a larger portion of the sample between the two resistance probes decomposes into ZnSb. Calculating the average temperature of the sample between the two probes from the change in electrical resistance would thus not be representative of the true complex temperature landscape of the sample. Furthermore, the average temperature between the resistance probes (a distance of 8–10 mm) is difficult to relate to the section illuminated by the X-rays (1 × 1 mm).

Real-space PDF refinements were performed to quantify the structural behavior leading up to the decomposition, and the corresponding unit-cell parameters and ADPs are shown in Fig. S5. From the sudden increase in the refined unit-cell parameters, a temperature increase of 145°C is estimated from Joule heating, which remains constant until the decomposition, as seen in the electrical resistance of the sample, Fig. 3 ▸(*a*). The subsequent behavior of the unit-cell parameters and ADPs correspond well to those obtained at 0.5 A mm^−2^ (a linear increase after initial Joule heating). This linear region extends to ∼40 mins, where electrical contact resistance in the interface between the β-Zn_4_Sb_3_ and ZnSb phases causes further heating in this specific region of the sample.

In the last experiment at 2.3 A mm^−2^, PXRD data were collected, Fig. S6. β-Zn_4_Sb_3_ is present in the very beginning but a significant peak shift towards lower angles indicates that severe Joule heating increases the sample temperature rapidly. The Joule heating is proportional to the square of the applied current, *i.e.* a fourfold and 16-fold increase compared with the first and second experiment (assuming equal absolute resistivity). Within seconds, a phase transition is seen from β-Zn_4_Sb_3_ to the high-temperature γ-Zn_4_Sb_3_ phase, which is stable above 493°C (Mozharivskyj *et al.*, 2004 ▸). This phase is present for two minutes before it decomposes into ZnSb because of migration of Zn out of the structure. This means that Zn is also mobile in γ-Zn_4_Sb_3_, but additional studies are needed to establish the migration mechanism for this transition. Nevertheless, simulation of the PDF of γ-Zn_4_Sb_3_ reveals the same correlation at *r* ≃ 2.7–2.9 Å as seen in both β-Zn_4_Sb_3_ and ZnSb (Fig. S7).

This high-current experiment emphasizes the difficulty of separating current and temperature effects because of the lack of independent control. Experimental measurement of the sample temperature is challenging since placing a thermocouple at the sample surface would give a cold-finger effect, and infrared cameras are imprecise. Independent control of temperature and current would enable a more detailed analysis of the origin of decomposition and we will attempt to improve this aspect in the next generation of the *operando* setup.

## 4. Conclusions   

In summary, the present study introduces *operando* measurements on TE materials by simultaneous collection of high-energy X-ray scattering and electrical resistance data on dense pellets subjected to electrical current. Three β-Zn_4_Sb_3_ samples were exposed to current densities of 0.5, 1.14 and 2.3 A mm^−2^, and both ion migration and decomposition reactions were quantified directly based on analysis of PXRD and PDF data. The *operando* setup also holds potential for studies of solid-state battery electrolytes or piezo and ferro-electric materials, for example.

## Supplementary Material

Supporting information. DOI: 10.1107/S205225251901580X/fc5041sup1.pdf
