# Supplementary material for: Operando X-ray scattering study of thermoelectric β-Zn4Sb3
Source: IUCrJ. 2020 Jan 1;7(Pt 1):100–4. doi: 10.1107/S205225251901580X (PMC6949592; doi:10.1107/S205225251901580X)
Supplement: Supplementary file 1 [file m-07-00100-sup1.pdf]

IUCrJ

Volume 7 (2020)

Supporting information for article:

Operando X-ray scattering study of thermoelectric β-Zn_4Sb_3

Lasse Rabøl Jørgensen, Christian Moeslund Zeuthen, Kasper Andersen Borup, Martin Roelsgaard, Nils Lau Nyborg Broge, Jonas Beyer and Bo Brummerstedt Iversen

S1. Rietveld refinement model

Two refinement models were employed and both are based on the β-Zn4Sb3 structure (ICSD #159090). The first model was used for sequential refinements of both PXRD and TS data, where the FullProf and PDFGui software were used in modeling, respectively. In this model, the site occupancy factors and atomic coordinates were kept fixed, and the isotropic ADPs were refined. The ADP for Sb1 and Sb2 were refined individually, whereas the ADP for all the zinc sites were constrained to be equal in an attempt to limit the number of free parameters. The reason for keeping the occupancy fixed is due to its severe correlation with the ADPs. Keeping the ADPs constant is undesirable since the current running through the material will cause significant Joule heating. Instead, the expected decrease in zinc occupancy from diffusion will be reflected in increasing ADPs, which will be decoupled from the effect of temperature by consulting the measured electrical resistance of the sample. The second refinement model is used in refinements of data acquired when the current was turned off and the temperature was constant, and for this reason, all ADPs are expected to be constant between the data sets and are therefore fixed. Instead, the site occupancy for the different zinc-sites was allowed to refine individually along with all possible atomic coordinates. These refinements were carried out in the JANA2006 software.

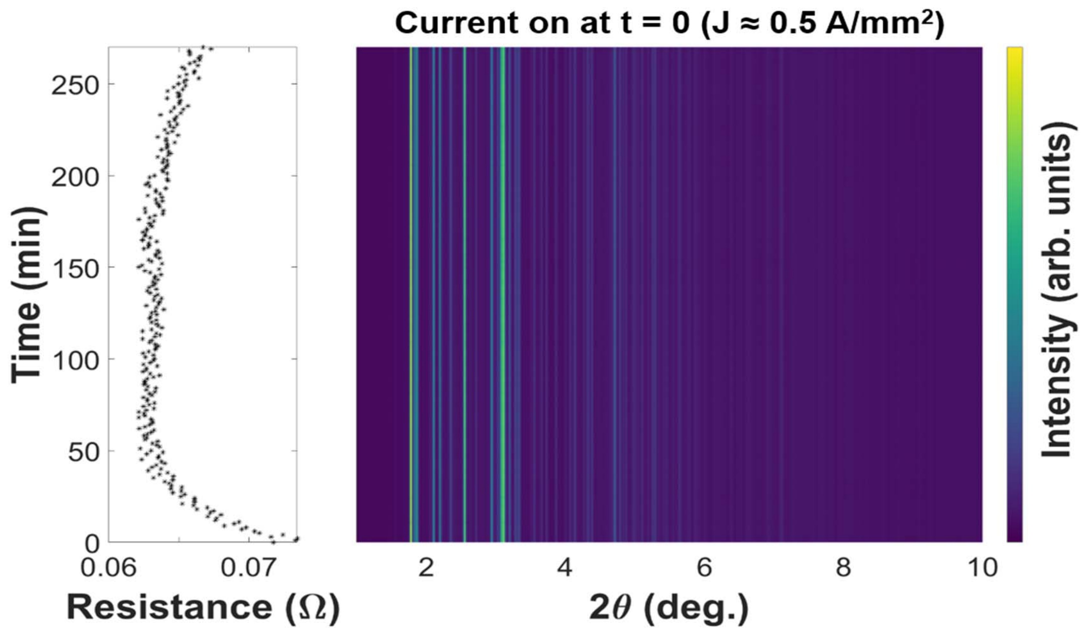

Figure S1 Surface plot of the PXRD patterns acquired during the experiment plotted alongside the electrical resistance. The applied current density is J = 0.5 A/mm^2.

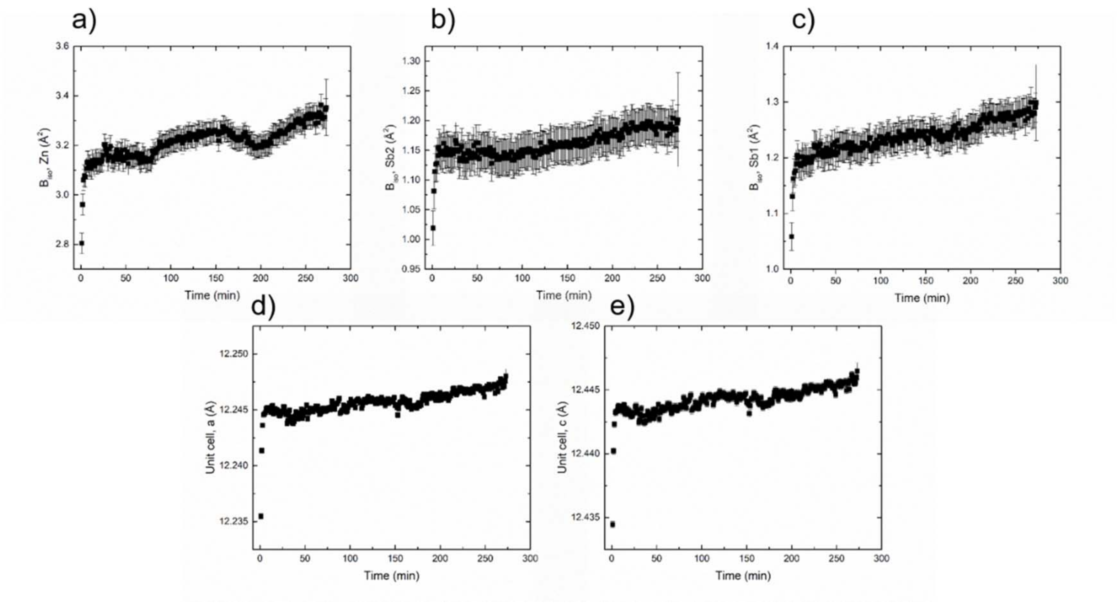

Figure S2 Extracted refinement parameters from a sequential Rietveld refinement. a)-c) shows ADPs from Zn1, Sb1 and Sb2. d)-e) shows the unit cell a and c parameters.

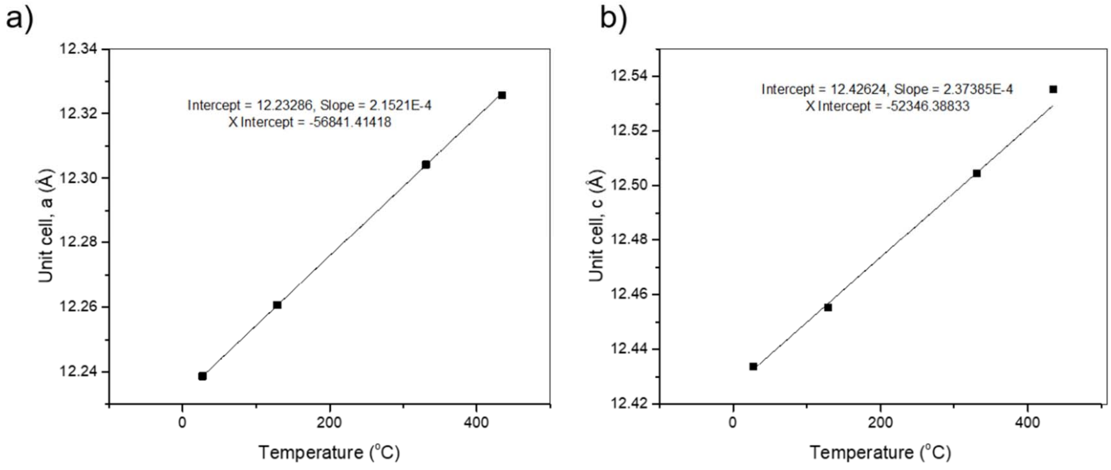

Figure S3 Thermal unit cell expansion coefficient of β-Zn_4Sb_3 for the a) a-axis and b) c-axis.

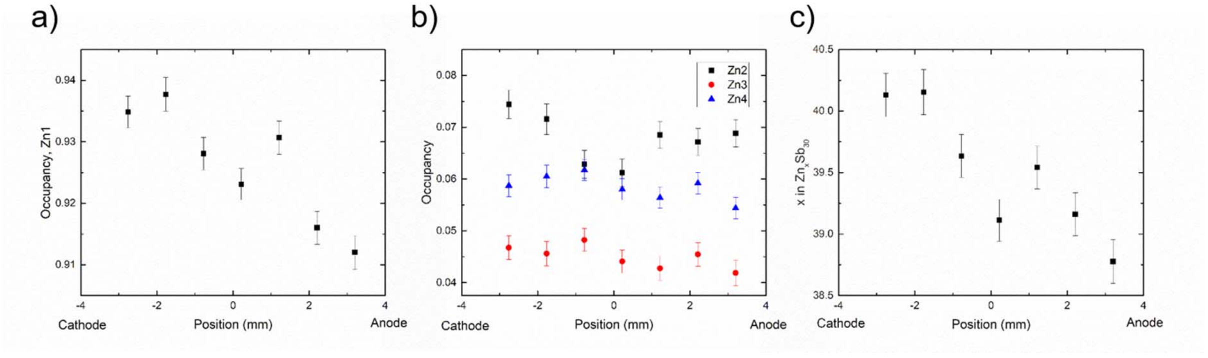

Figure S4 The extracted occupancy of the a) Zn1 site and b) Zn2, Zn3 and Zn4 sites as a function of sample position. C) Shows the final composition.

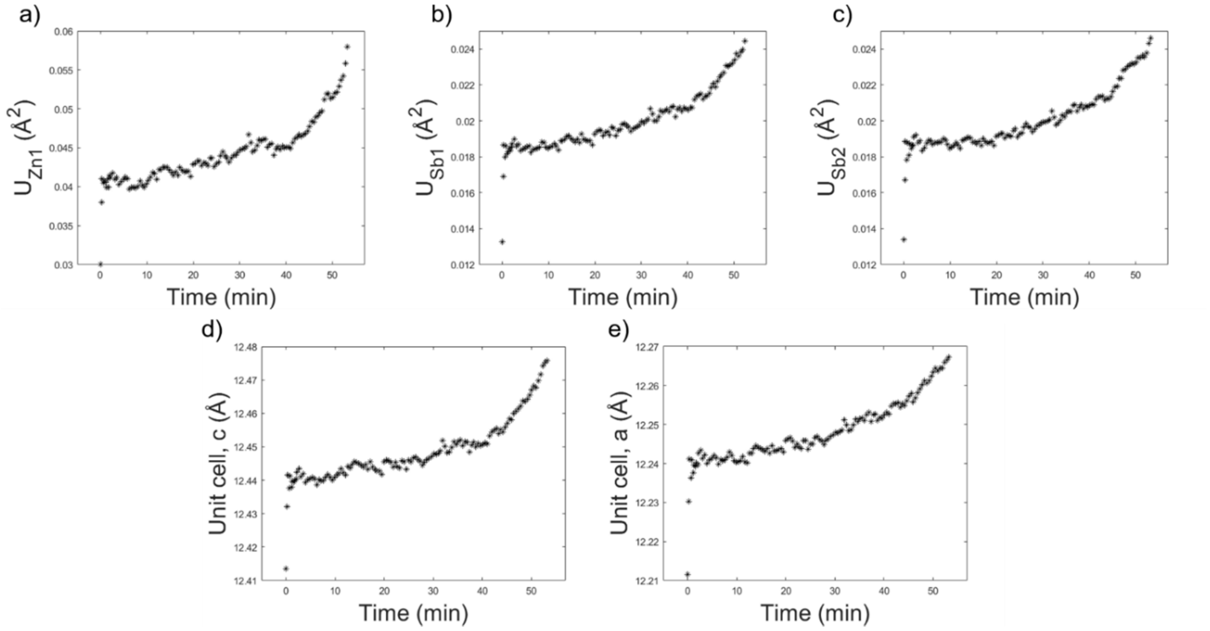

Figure S5 Extracted refinement parameters from a sequential Rietveld refinement. a)-c) shows ADPs from Zn1, Sb1 and Sb2. d)-e) shows the unit cell a and c parameters.

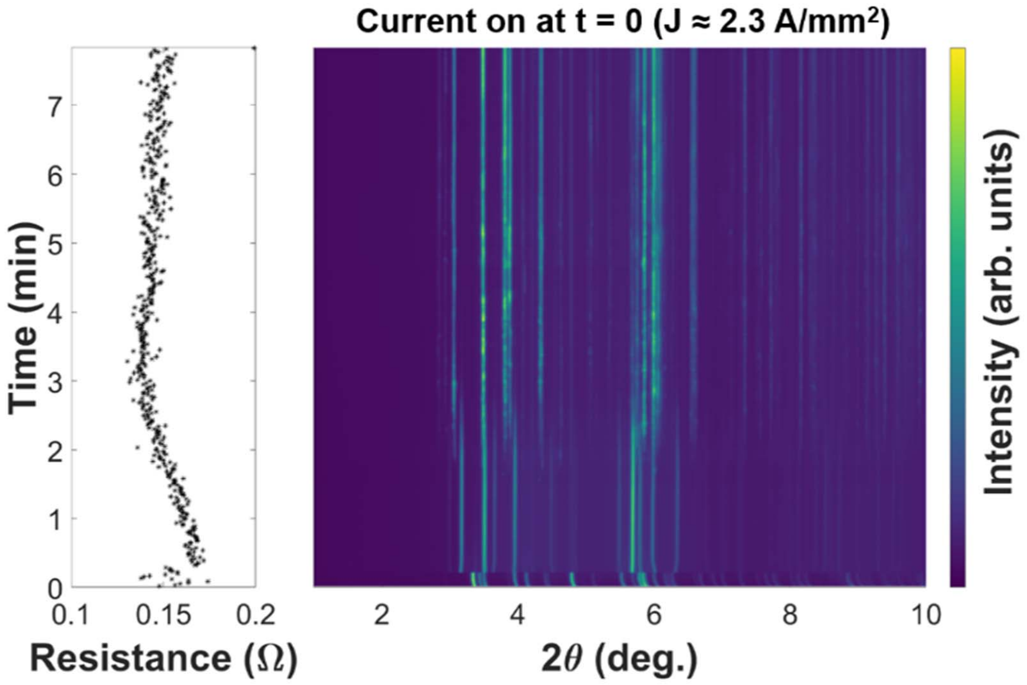

Figure S6 Surface plot of PXRD patterns obtained during the experiment plotted alongside the electrical resistance. A current density of J = 2.3 A/mm2 was applied at t = 0.

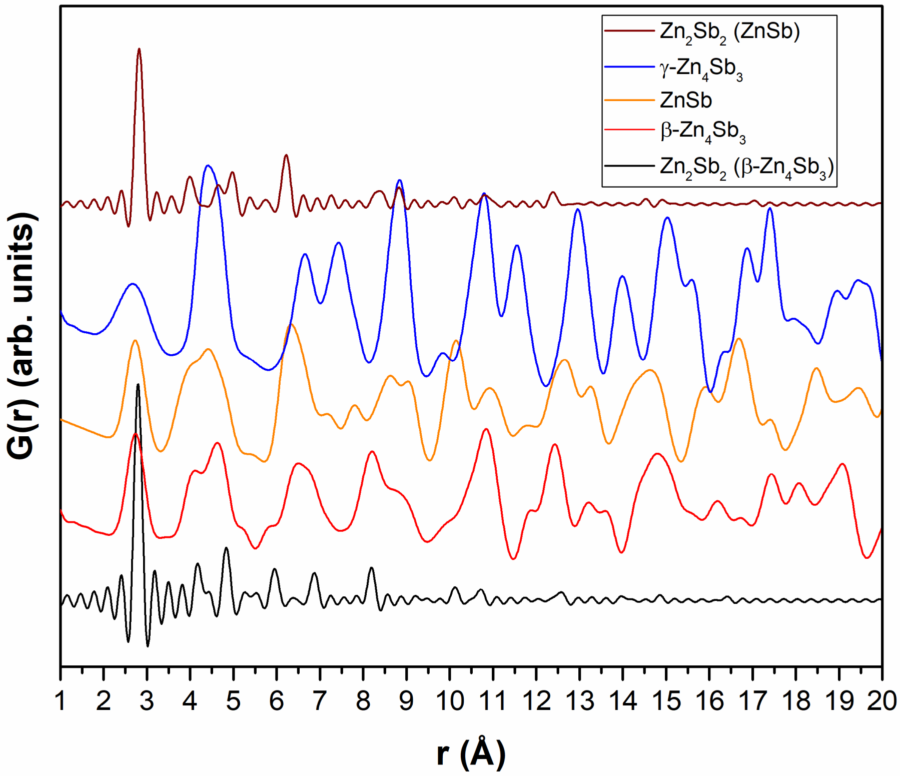

Figure S7 Simulated Pair Distribution Functions for the rhomboid unit Zn2Sb2 found in β-Zn_4Sb_3 (bottom), β-Zn_4Sb_3, ZnSb, γ-Zn_4Sb_3 and the rhomboid unit Zn2Sb2 found in ZnSb (top).
